# Examining the role of phonological and semantic mechanisms during morphological processing of sentences in 7-year-old children

**DOI:** 10.1093/cercor/bhaf115

**Published:** 2025-05-14

**Authors:** Marjolein Mues, Avantika Mathur, James Booth

**Affiliations:** Department of Psychology and Human Development, Vanderbilt University, Hobbs Hall, 1818 DeFord Bailey Ave, Nashville 37203, TN, United States; Department of Psychology and Human Development, Vanderbilt University, Hobbs Hall, 1818 DeFord Bailey Ave, Nashville 37203, TN, United States; Department of Psychology and Human Development, Vanderbilt University, Hobbs Hall, 1818 DeFord Bailey Ave, Nashville 37203, TN, United States

**Keywords:** fMRI, language, morphology, phonology, semantics

## Abstract

Morphology refers to the smallest difference in sound that makes a difference in meaning, such as *walk* versus *walked*. Morphological skill is a key linguistic feature that impacts language and literacy outcomes, but its neural underpinnings have mostly been examined at the word level. We examined if phonological and semantic mechanisms play a role during morphological processing in sentences in 7-year-old children using functional MRI. Using a novel functional localizer approach that correlates brain activation during sound and meaning in-scanner tasks with standardized scores for phonology and semantics, we show that morphological processing is especially reliant on phonological mechanisms given significant activation in the left dorsal inferior frontal gyrus and left posterior superior temporal gyrus. Semantic mechanisms were engaged to a lesser degree in the left ventral inferior frontal gyrus. Exploratory whole-brain analyses revealed a brain–behavior correlation in the cerebellum showing that greater activation during morphological processing was related to lower language abilities. Our results suggest that processing morphological structures in sentences relies mostly on phonemic segmentation, and that those with lower language may compensate for their lower phonological skill by engaging the cerebellum to amplify and refine those phonemic representations to aid in segmentation when listening to sentences.

Auditory sentence processing relies on the integration of a variety of linguistic information sources that aid the listener in achieving successful comprehension (Lee and Watson 2012). One such information source is morphological structure. Morphology refers to study of the smallest units of language that carry meaning. For example, the morpheme/-*s* in the word *cats* indicates that the word is plural and the morpheme/-*ed* in *walked* signals the past tense of the verb. With the encoding of such information, these morphological structures provide cues that help in understanding word and sentences.

Given morphology is the foundational building block of single words, previous studies have frequently examined auditory processing of this information at the lexical level. Such studies show that morphological processing of words rely on both phonological and semantic mechanisms (Leinonen et al. 2009; Schwarz et al. 2023). This is perhaps unsurprising as morphemes need to be phonemically decoded in a speech stream after which their meaning can be determined. The weight that semantic and phonological mechanisms carry during morphological processing seems to vary between individuals. For example, a recent study showed that semantic engagement was especially strong during word processing of morphologically complex words for individuals who showed less effective phonological processing efficiency. These individuals also showed a slower response time on the morphological processing task, indicating that semantic mechanisms may not be as efficacious for morphological processing as phonological mechanisms (Schwarz et al. 2023). This is in line with Friederici’s sentence processing theory that assumes phonological processing occurs first and semantic analyses occur at a later stage, even after syntactic analyses (Friederici 2002; Friederici 2011), implying that individuals who rely more on phonological mechanisms for morphological processing at an earlier stage are more effective at sentence processing compared to those who rely on later-occurring semantic mechanisms. Moreover, children with developmental language disorder (DLD) that experience difficulties with phonological processing, but have relatively intact semantic knowledge, show difficulties in morphological processing (Georgiou and Theodorou 2023; Calder et al. 2024), possibly because they cannot rely on faulty phonological mechanisms so need to engage to a greater degree semantic knowledge to process morphological cues. To the best of our knowledge, research has not yet examined this hypothesis in DLD, although it has been demonstrated that word reading of morphologically complex words was facilitated by overreliance on semantic processes in the presence of a phonological deficit in children with dyslexia (van der Kleij et al. 2019).

A later role for semantics is also in accordance with two main theories of morphological processing: the Distributed Morphology Framework (DMF) and the Connectionist Morphology Framework (CMF). DMF states that words are first decomposed into their constituent morphemes that are first processed syntactically and then semantically (Harley and Noyer 2003). This model poses that words and sentences are processed in the same way: they are syntactically decomposed to the smallest level of individual morphemes that are then attributed meaning (Embick and Noyer 2007). Within the CMF, however, morphemes are viewed as the interaction of form (phonological information) and meaning (semantic information), reflecting systematic relationships between the two (Gonnerman et al. 2007). Here too, semantic information is processed later, as form first needs to be processed before meaning can be attributed, underscoring the heavier reliance on phonological mechanisms earlier in processing, but relationships between form and meaning can differ depending on whether an individual is tasked with single words or higher-level structures such as sentences. Previous studies have shown that the encoded information within morphological structures seems to be more influential for auditory sentence processing than for single-word processing (Allen et al. 2003; Leminen et al. 2016), indicating that sentence-level morphological processing may not follow the same principles as single-word morphological processing.

As humans are generally tasked with processing full sentences rather than single words, examining morphology in the context of sentences is more ecologically valid. As with words, multiple cognitive processes need to be integrated to successfully process and ultimately comprehend sentences, including semantic and phonological information (Friederici 2002). Processing morphological violations in auditorily presented sentences engages the left inferior frontal and temporal cortex (Cooke et al. 2006). Word-level MRI studies, both functional and diffusion, underscore the involvement of a left fronto-temporal language network through dorsal and ventral streams of processing (Tyler et al. 2005; Lehtonen et al. 2006; Kovelman et al. 2008; Yablonski et al. 2021). According to dual stream theories, the dorsal pathway is more crucially related to phonological processing, whereas the ventral pathway is related to semantic processing (Hickok and Poeppel 2004; Hickok and Poeppel 2007). The existence of this language network has been well established in adults (Vigneau et al. 2006) and replicated across various languages (Malik-Moraleda et al. 2022). Nine-year-old children already show the presence of a left fronto-temporal language network that is recruited during phonological and semantic tasks (Wang et al. 2023). Specifically, these children show increased activation in the left dorsal inferior frontal gyrus (IFG) and left posterior superior temporal gyrus (pSTG) during a phonology task compared to a semantic task and increased activation in the left posterior middle temporal gyrus (pMTG) for the opposite contrast (Wang et al. 2023). Children as young as 5 to 7 years old also show this specialization for semantics and phonology processing in the temporal lobe, specifically the left pSTG for phonological tasks and the left pMTG for semantic tasks, but not yet in the frontal lobe (Weiss et al. 2018; Mathur et al. 2020; Wang et al. 2021).

Previous behavioral research shows that both phonology and semantic knowledge are important for morphological processing during auditory sentence processing. However, it is unclear how these neural mechanisms are engaged on-line during sentence processing. We chose to examine young children (7-year-olds) because there are large individual differences in language skill in this age range. We used a novel localizer approach to independently identify brain areas involved in phonological and semantic processing by correlating activation during sound and meaning tasks with standardized measures of phonological and semantic skill. Then, we examined how these brain regions are engaged during the grammaticality judgments of auditory sentences with morphological violations and how their engagement is related to individual differences in standardized measures of general language and morphological skill. We hypothesized that auditory sentence processing should be associated with robust engagement of phonological mechanisms when making grammaticality judgments and that higher skill should be associated with a more reliance on these mechanisms, resulting in greater activation in the left dorsal IFG and the left pSTG. We also hypothesized that auditory sentence processing should be associated with engagement of semantic mechanisms, but that lower language skills should be associated with a more reliance on these mechanisms, resulting in greater activation in the left ventral IFG and the left pMTG.

## Method

The hypotheses and analysis plans of this study were preregistered through the Open Science Framework after verifying participant eligibility criteria and prior to data analyses. The preregistration is available at https://osf.io/juzcm.

### Participants

Participants were selected from an open-access dataset named “A longitudinal neuroimaging dataset on language processing in children ages 5, 7, and 9 years old,” available on OpenNeuro (https://openneuro.org/datasets/ds003604/versions/1.0.7) (Wang et al. 2022). The dataset contains longitudinal data at three time points: when children were 5 years old, 7 years old, and 9 years old. This study employs data from the second time point when children were ~7 years old. This time point initially included 294 participants. After applying motion and accuracy criteria (see Section 2.3.1) for the three tasks of interest (morphology, phonology, and semantics), 105 children remained. Finally, after checking behavioral inclusion criteria, a sample of 92 participants were included in the analysis. The behavioral inclusion criteria were (1) mainstream English speakers, defined as scoring 9 out of 15 on the Diagnostic Evaluation of Language Variation Part 1 Language Variation Status (Seymour et al. 2005); (2) primarily right-handed defined as performing at least three out of five tasks out of writing, drawing, picking up, opening, and throwing items using their right hand; (3) a score of > 70 on the Kaufman Brief Intelligence Test, Second Edition (KBIT-2) (Kaufman 2004); and (4) no clinical diagnosis of neurological, psychiatric, or developmental disorders as reported in a parent questionnaire.

Included participants (37 male, 55 female) were between 7.0 and 8.3 years old (*M* = 7.4, *SD* = 0.3) at the time of testing. Participating families completed informed consent and assent forms prior to testing. A list of subject IDs for the participants included in this study can be found on Github (https://github.com/marjoleinmues/Phonological_Semantic_Mechanisms_During_Morphological_Processing_7yo) to enhance reproducibility. The study procedures were approved by the Institutional Review Board of the University of Texas, Austin (protocol number 2014-07-0018).

### Behavioral assessments

#### Phonological processing

The elision subtest of the Comprehensive Test of Phonological Processing, second edition (Wagner et al. 1999), was used to examine behavioral phonological processing. Elision measures the ability to omit one or more phonemes from a word. The child is, for example, asked: “say the word *cat without* c.” Participants’ scaled scores on the elision subtest in combination with a phonological functional MRI (fMRI) task were used to create a functional localizer for further analysis.

#### Semantic knowledge

Semantic knowledge was measured with the word classes subtest of the Clinical Evaluation of Language Fundamentals, fifth edition (CELF-5) (Wijg et al. 2013). In this task, the child is asked to indicate which two out of three or four pictures “go together.” For example, the child sees pictures of milk, an apple, and a banana and is supposed to decide that the apple and the banana go together because they belong to the same semantic category of fruit. The scaled scores on the word classes subtest in combination with a semantic fMRI task were used to create a functional localizer for further analysis.

#### Language abilities

We included two standardized measures in our brain–behavior analyses to examine if the role that phonological and semantic mechanisms play during sentence processing is related to general language ability or morphological skill. General language ability was characterized by the sentence repetition subtest of the CELF-5 (Wijg et al. 2013). We chose sentence repetition as a proxy of general language ability as it is perhaps the best reflection of various underlying linguistic processes at different levels including speech perception, lexical knowledge, and grammatical encoding (Klem et al. 2015) and does not disproportionally rely on either phonological or semantic skills. Morphological skill was measured with the word structure subtest of the CELF-5. This task elicits different morphemes by asking questions such as “here is one girl, here are two …?”. The child is then supposed to answer with the target word and add the correct morpheme, in this case answering: “girl*s*.”

#### Nonverbal cognitive abilities

The KBIT-2 (Kaufman 2004) was used to assess nonverbal IQ. All children in the final sample scored greater than or equal to a standard score of 70. Nonverbal cognitive abilities were included in our analyses as a control variable.

### fMRI tasks

Prior to their fMRI visit, children were invited for a practice session in a mock scanner to get familiar with the in-scanner tasks and the scanning environment. During their actual MRI visit, T1-weighted and task-based fMRI sequences were collected. Data for four fMRI tasks were collected, three of which were analyzed for this paper: a grammaticality task, a sound task, and a meaning task. For each of these tasks, included participants completed two runs. Participants typically completed all four tasks (eight runs) in two scanner days, with each session lasting around an hour. Participants were able to respond to stimuli through button boxes and were instructed to respond as quickly and accurately as possible by using their right index finger to respond “yes” and their right middle finger to respond “no.” The experimental tasks have previously been described in Wang et al. (2022).

The grammaticality task consisted of an auditory sentence judgment task. Children were asked to indicate if “the way she speaks sounds right,” eliciting a yes or no response. The task had three experimental conditions: a grammatically correct condition, a plurality violation condition, and a finiteness violation condition. Children also completed a perceptual control condition where they heard two frequency-modulated white noise sounds and were instructed to press the yes button. Table 1 shows example sentences of each condition. Each of the two trials contained 40 runs (ie 80 trials in total), with 10 trials per condition per run. Stimuli duration ranged between 2,740 and 4,972 ms and was followed by a 1,234- to 4,611-ms jittered response interval. The length of trials (5,174 to 8,422 ms) was equated across conditions. In this study, we compare the finiteness violation condition with the grammatically correct condition to characterize morphological processing.

**Table 1:** Examples of grammaticality task.

Condition	Example	Description
Grammatically correct	She is moving the box	No grammatical violation
Plurality violation	Every day, they stir three pot*	A violation in number and object agreement
Finiteness violation	Every day, she **press** one button*	A morphological violation on the verb form
Perceptual control	Shh-shh	Frequency-modulated noise

The sound task consisted of an auditory word-level phonological judgment. Participants heard two sequentially auditory one-syllable words and had to indicate whether the two words “shared any sounds,” eliciting a yes or no response. The task had three experimental conditions: a rhyme condition, an onset condition where only the first sound was shared between the two words, and an unrelated condition; see Table 2 for example stimuli. The children also completed the perceptual control condition as described before. The task included 48 trials per run (ie 96 runs in total), with each run containing 12 trials per condition and lasting ~3 min. Each word had a duration ranging from 439 to 706 ms, and the second word was presented ~1,000 ms after the onset of the first word. The total stimuli duration per trial ranged from 1,490 to 1,865 ms and was followed by a jittered response interval that varied between 1,500 and 2,736 ms.

**Table 2:** Examples of phonology and semantic task.

Condition	Example	Description
*Sound task*		
Rhyme condition	Wide-Ride	Two words that share the coda
Onset condition	Coat-Cup	Two words that share the first sound
Unrelated condition	Zip-Cone	Two words that do not share sounds
Perceptual control	Shh-shh	Frequency-modulated noise
*Meaning task*		
High association	Water-Drink	Two words with a strong semantic association
Low association	Syrup-Pancake	Two words with a weak semantic association
Unrelated condition	Flush-Cliff	Two words with no semantic association
Perceptual control	Shh-shh	Frequency-modulated noise

The meaning task consisted of an auditory word-level semantic judgment. Just as for the sound task, participants would hear two sequentially presented one-syllable words, but for this task they were asked if the words “go together.” The task had three experimental conditions: a high association condition, a low association condition, and an unrelated condition; see Table 2 for examples. Children also completed the perceptual control task. The semantic task included 48 trials per run (96 runs in total), with each run containing 12 trials per condition and each run lasting ~3 min. Each word had a duration ranging from 500 to 700 ms, and the second word was presented ~1,000 ms after the onset of the first word. The total stimuli duration per trial ranged from 1,500 to 1,865 ms and was followed by a jittered response interval that varied between 1,800 and 2,701 ms. More specifics of the fMRI tasks can be found in Wang et al. (2022).

#### Accuracy and motion criteria

Only participants with acceptable task accuracies and who did not show a response bias were included in the analyses to ensure that all included participants were engaged during the task. Acceptable accuracy was defined as a score of >50% correct on the perceptual control task and >40% correct on the “easiest” condition for each of the experimental tasks. The following conditions were considered the easiest (see also Table 3 for sample accuracies): for the grammaticality task, the plurality violation condition; for the sound task, the rhyming condition; and for the meaning task, the high association condition. Response bias was indicated by an accuracy difference <40% between the rhyme and unrelated conditions for phonology, the high association and unrelated conditions for semantics (word level), and between the plurality violation and the grammatically correct conditions for the grammaticality task (sentence level).

**Table 3:** Percentage correct per condition per fMRI task.

*M*		*SD*
*Grammaticality task*		
Grammatical	81.4	14.5
Finiteness violation	71.6	21.3
Plurality violation	88.5	12.1
Perceptual control	95.5	8.7
*Sound task*		
Rhyme	88.4	12.4
Onset	69.8	16.6
Unrelated	84.8	12.7
Perceptual	95.3	7.0
*Meaning task*		
High association	89.6	10.3
Low association	84.4	14.1
Unrelated	83.7	14.1
Perceptual control	95.9	6.9

Acceptable motion was defined as participants having no more than 10% of all volumes or six consecutive volumes being outliers per run. Outlier volumes were volumes exceeding 1.5 mm volume-to-volume head movement in any direction, >5 mm from the mean functional image or the baseline image (first functional image), or deviations of >4% from the global mean signal.

#### fMRI data acquisition

Images were acquired using 3 T Siemens Skyra MRI scanner with a 64-channel head coil. Functional images were acquired using a susceptibility T2-weighted single-shot EPI method with the following parameters: TR = 1,250 ms, TE = 30 ms, FOV = 256 × 256 mm, matrix size = 128 × 128, bandwidth = 1,776 Hz/Px, slice thickness = 2 mm without gap, number of slices = 56, voxel size = 2 mm isotropic, flip angle = 80°, and multiband acceleration factor = 4. Slices were acquired interleaved from foot-to-head. A high-resolution T1-weighted structural image was also acquired using the following parameters: TR = 1,900 ms, TE = 2.43 ms, FOV = 256 × 256 mm, matrix size = 256 × 256, bandwidth = 180 Hz/Px, slice thickness = 1 mm, number of slices = 192, voxel size = 1 mm isotropic, and flip angle = 9°.

### Data analysis

Preprocessing and analyses were analyzed using Statistical Parametric Mapping, 12th edition, software (https://www.fil.ion.ucl.ac.uk/spm/software/spm12/).

#### fMRI preprocessing and first-level analysis

For preprocessing, all functional images were first realigned to their mean functional image across runs. Then, using the CerebroMatic toolbox that contains anatomical estimates generated from 1,919 participants spanning an age range between 13 months and 75 years, anatomical images were segmented and warped to a pediatric template of children between 7.0 and 9.0 years old (Wilke et al. 2017). This was done by applying an anatomical brain mask created from gray matter, white matter, and cerebrospinal fluid to the original anatomical image to create a skull-stripped image. All functional images and the mean functional image were co-registered to this skull-stripped anatomical image and normalized to the pediatric template using a 6-mm Gaussian kernel.

To reduce motion effects, we used ArtRepair (https://www.nitrc.org/projects/art_repair/) to identify outlier volumes in the functional images (Mazaika et al. 2009). Outlier volumes were defined as volumes exceeding 1.5 mm volume-to-volume head movement in any direction, head movement >5 mm in any direction from the mean functional image across runs, or the baseline image (first functional image), or deviations of >4% from the global mean signal. Outlier volumes were replaced with interpolated values from adjacent non-outlier volumes. No more than 10% of volumes in each run and no more than six consecutive volumes for any individual were interpolated in this way as participants with more outlier volumes were excluded from the analyses. These criteria are based on previous publications using the same data (eg Wagley and Booth 2021; Wang et al. 2021).

First-level statistical analyses were run per task (grammaticality task, sound task, and meaning task) and included 10 regressors for each run, one for each of the task conditions (ie rhyme, onset, unrelated, and perceptual condition for the sound task) and six motion regressors that were estimated in the realignment step. Repaired volumes were de-weighted (Mazaika et al. 2009). All experimental trials (correct and incorrect) were included in the analysis and modeled using a canonical hemodynamic response function.

#### Anatomical regions of interest

Four anatomical regions were chosen based on previous literature (eg Mathur et al. 2020; Wang et al. 2023), specifically the left pMTG (*y* = −39), the left pSTG (*y* = −26), the dorsal left inferior frontal gyrus opercularis (opIFG), and the ventral left inferior frontal gyrus triangularis (trIFG). These regions were identified using the anatomical automatic labeling (AAL) atlas template (Rolls et al. 2020) using the AFNI-3dcalc command (https://github.com/afni/afni/blob/master/src/3dcalc.c). Because the AAL atlas is based on the adult brain, the T1 structure of the AAL atlas was warped to pediatric T1 template before selecting the anatomical Regions of Interest (ROIs).

#### Functional regions of interest

Within the anatomical regions, functional localizers for phonology (pSTG and opIFG) and semantics (pMTG and trIFG) were created. The MarsBar toolbox (Brett et al. 2002) was used to identify the top 500 voxels (regardless of significance) within the anatomical regions for the contrasts of interest. For the sound task, the contrast of interest consisted of onset + rhyme > perceptual correlated with the behavioral score on the elision task, and for the meaning task, the contrast of interest consisted of high + low association > perceptual correlated with the behavioral score on the word classes task. By creating functional localizers using both brain and behavior data, we expect to reliably localize voxels that are functionally coherent with phonological and semantic processing. The top 500 voxels associated with the respective phonological and semantic contrasts within the pSTG, opIFG, pMTG, and trIFG constituted our four functional ROIs within which analyses were performed. A visualization of the four functional ROIs is shown in the Results section.

#### Analytical approach

**Preregistered analyses**. To examine if there is activation in the phonological and semantic ROIs during morphological processing, we created a first-level contrast for the finiteness violation > grammatical condition for the grammaticality task. We then examined if there were any voxels for this contrast that were significantly activated within each of the phonological and semantic ROIs. Using 3dClustSim, 10,000 Monte Carlo simulations of random noise activations using a corrected cluster-wise probability threshold of < 0.05 and a voxel-wise height threshold of 0.005 (uncorrected) were performed in each ROI. The number of simulations in which clusters of different sizes appear within each ROI was tallied, and these tallies were used to calculate the voxel cluster size needed for significance for a given ROI. Clusters exceeding these size thresholds were considered significant. Based on this calculation, a cluster size of 11, 11, 12, and 15 voxels was needed for significance within the top 500 voxel masks of the left pSTG, opIFG, pMTG, and trIFG, respectively (AutoCorrelation Function (ACF) values = 0.45, 4.58, 12.24). Clusters exceeding these cluster sizes were significantly activated during the grammaticality task.

We further performed correlation analyses examining positive and negative correlations between behavioral language scores (sentence repetition and word structure) and the grammaticality contrast (finiteness violation > grammatically correct condition) within each ROI. These analyses were performed in SPM to examine if clusters of activation would be associated with this correlation. Here too, the 3dClustSim cluster size thresholds for significance per ROI were used to identify significant clusters. Correlation analyses were separately performed for sentence repetition as an index of general language abilities and word structure as an index of morphological skill.

**Exploratory analysis.** In addition to the preregistered analyses, we conducted an exploratory whole-brain correlation. We repeated the correlation analyses between behavioral language (sentence repetition and word structure) and the grammaticality task (contrast: finiteness violation > grammatically correct condition) for the whole brain. 3dClustSim was used with a corrected cluster-wise probability threshold of < 0.05 and a voxel-wise height threshold of 0.005 (uncorrected) to determine the size threshold needed for significance. This calculation indicated that clusters needed to be at least 236 voxels to reach significance (ACF values = 0.45, 4.58, 12.24). Correlation analyses were conducted separately for sentence repetition and word structure tasks.

## Results

### Behavioral results

For the in-scanner tasks, children had an average accuracy of 84.3%, 84.6%, and 88.4% for the grammaticality task, sound task, and meaning task, respectively. Mean accuracies for each condition per task (collapsed across both runs) are summarized in Table 3. Behavioral test scores for phonology, semantics, nonverbal cognitive abilities, and language are reported in Table 4.

**Table 4:** Standardized test scores.

*M*		*SD*	Range
*Phonology*			
Elision^a^	11.4	2.8	5 to 17
*Semantics*			
Word classes^a^	12.5	3.2	3 to 19
*Nonverbal cognitive abilities*			
KBIT^b^	111.2	16.6	74 to 147
*Language*			
Sentence repetition^a^	11.7	3.1	6 to 19
Word structure^a^	10.2	2.4	6 to 17

^a^Scaled scores (population mean of 10, *SD* of ±3)

^b^Standard scores (population mean of 100, *SD* of ±15).

### Preregistered analyses

#### Top 500 voxels for phonology and semantics

**Fig. 1:**
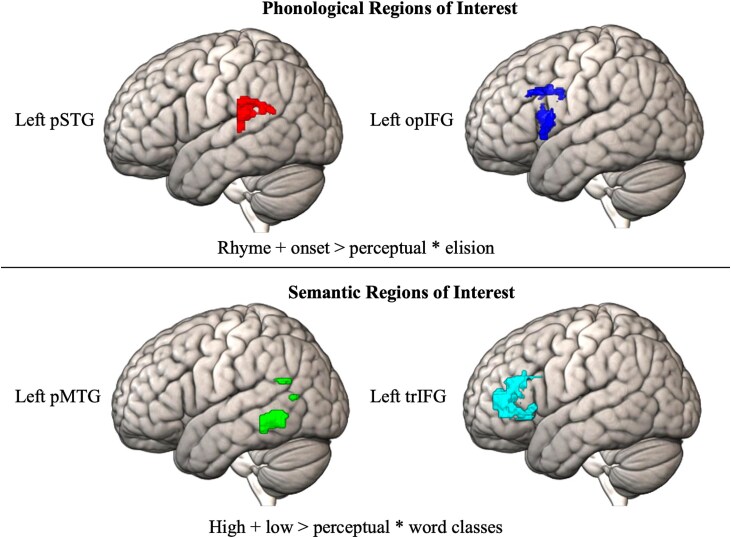
Top 500 voxels for sound and meaning tasks. Activation during the sound and meaning tasks was correlated with the behavioral tasks for phonology and semantics (elision/word classes). The top 500 activated voxels for this contrast within each of the anatomical regions of interest were extracted. Anatomical regions of interest were the left posterior superior temporal gyrus (pSTG) and left inferior frontal gyrus opercular (opIFG) for phonological processing and the left posterior middle temporal gyrus (pMTG) and left inferior frontal gyrus triangularis (trIFG) for semantic processing. These top 500 voxel masks then constituted our functional localizers for phonological (pSTG and opIFG) and semantic (pSTG and trIFG) processing.

As described in the Methods, we used the sound and meaning fMRI tasks correlated with standardized scores to identify the top 500 voxels associated with phonological processing in the pSTG/opIFG and with semantic processing in the pMTG/trIFG. Fig. 1 visualizes the top 500 voxels showing maximal activation (regardless of significance) associated with the sound and meaning contrasts in the anatomical regions. These top voxels constituted our four functional ROIs.

#### Activation in phonological and semantic ROIs during morphology task

**Fig. 2:**
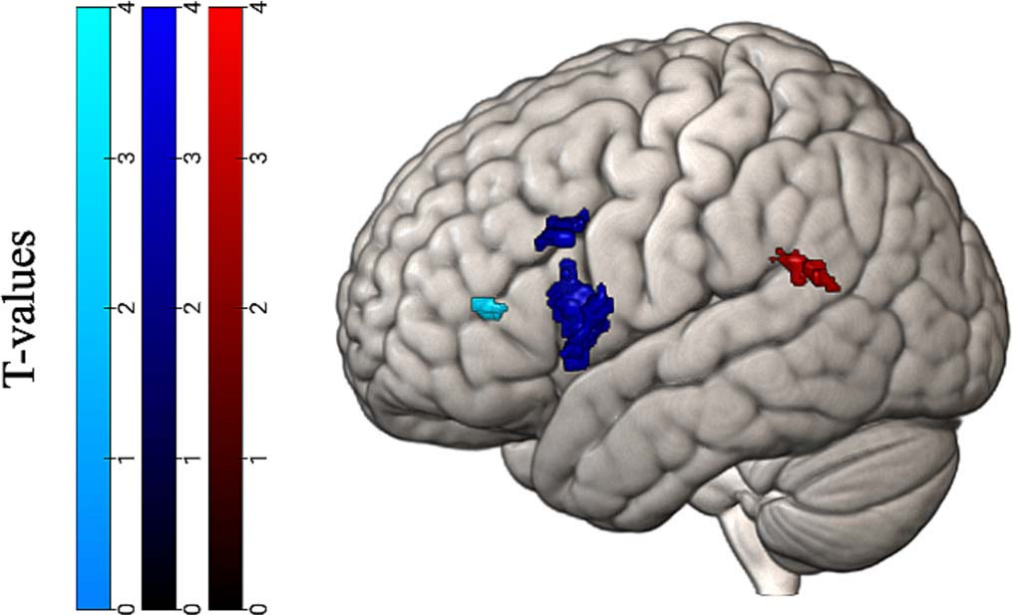
Significant clusters of voxels during the grammaticality task (finiteness > grammatically correct contrast) in the phonological and semantic functional localizers. Significant clusters in the phonological regions are located in the pSTG, depicted on the right, and opIFG in the middle. Significant clusters in the semantic regions are in the trIFG, depicted on the left.

To answer our main research question focused on whether phonological or semantic mechanisms underlie morphological processing, we examined activation during the grammaticality task for the contrast finiteness violation > grammatically correct within the ROIs for the sound and meaning task. The analyses revealed significant clusters in the left pSTG, opIFG, and trIFG, but not the left pMTG. Significant clusters are visualized in Fig. 2 and reported in Table 5.

**Table 5:** Cluster information for the grammaticality task—ROIs.

Anatomical location	BA	MNI coordinates			*k*	*Z* value
		*X*	*Y*	*Z*		
Left pSTG	39	−64	−50	20	25	3.33
	39	−46	−44	24	24	3.25
Left opIFG	44	−38	14	10	278	4.92
	44	−56	18	32	81	3.47
Left trIFG	46	−32	38	10	18	3.25

Note: Approximate Brodmann areas (BA), MNI coordinates of cluster peaks, cluster size (*k*), and *Z* values. These correspond to the regions in Fig. 2.

#### Correlations with general language abilities and behavioral morphological abilities within ROIs

**Fig. 3:**
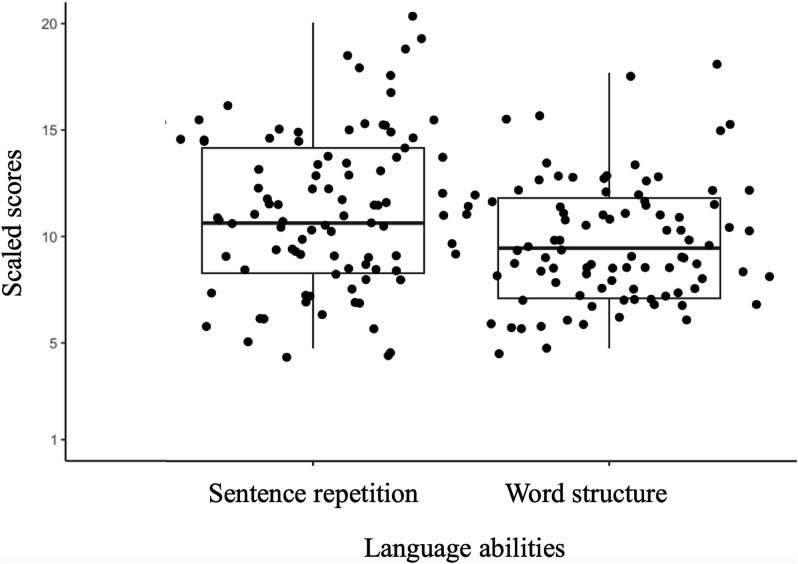
Boxplots showing the distribution of scores for sentence repetition and word structure subtests of the CELF-5 for all participants. The *y*-axis shows all possible scores. Scores are standardized with a mean of 10 and a standard deviation of 3.

Our data did not show significant positive or negative correlations between activation during the grammaticality task (finiteness > grammatically correct contrast) and standardized language abilities as measured with the sentence repetition or word structure task in the four ROIs. Fig. 3 shows boxplots of the distribution of scores for sentence repetition and word structure along a *y*-axis of all potential scores (1 to 20).

### Exploratory analyses

**Fig. 4:**
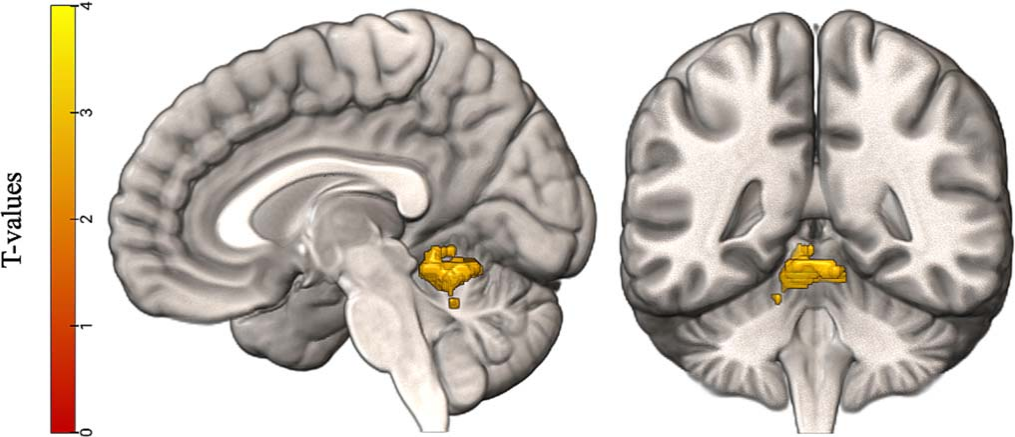
Results of the exploratory whole-brain analyses. Visualized is a significant cluster in the grammaticality task (finiteness > grammatical) that showed a negative correlation with general language abilities as measured by the sentence repetition subtest on the CELF-5. Using the SUIT atlas, clusters were determined to be in the left and right anterior lobe of the cerebellum (lobule I to IV, see Table 6).

We conducted exploratory whole-brain correlation analyses between the grammaticality task (finiteness > grammatical) and both sentence repetition and word structure. The results did not show significant results for the word structure task, but we did find a cluster exceeding the significance threshold for a negative correlation with sentence repetition. The significant cluster was in the cerebellum and showed three peaks. We used the SUIT atlas, a probabilistic atlas of the cerebellum confined to the MNI152 space (Diedrichsen 2006), to identify the anatomical area of the peaks. The atlas showed that two out of three peaks were in the left anterior lobe and one in the right anterior lobe; see Fig. 4. Specific information about the cluster and its three peaks is shown in Table 6.

**Table 6:** Cluster peak information for significant whole-brain correlation in the cerebellum.

Area as defined by SUIT atlas		MNI coordinates			*k*	*Z* value
*X*			*Y*	*Z*		
Peak 1	Left lobule I to IV	−6	−50	−14	294	4.98
Peak 2	Left lobule I to IV	−8	−42	−10		3.66
Peak 3	Right lobule I to IV	6	−46	−14		3.44

Note: the SUIT atlas is a probabilistic atlas of the cerebellum (Diedrichsen 2006).

## Discussion

The aim of this study was to examine the underlying neural underpinnings of morphological processing in sentences in 7-year-old children. Based on prior literature at the word level (eg Leinonen et al. 2009; Schwarz et al. 2023), we hypothesized that morphological processing would rely on phonological and semantic mechanisms. We examined this hypothesis by using a novel functional localizer approach that used brain-behavioral correlations within anatomical regions of interest. We defined these regions of interest by correlations of phonological awareness with activation during a sound task in the dorsal IFG and pSTG and of a semantic judgment with activation during a meaning task in ventral IFG and pMTG. This approach ensured that our functional regions of interest were related to either phonological processing or semantic processing. We then examined how these regions were engaged during morphological processing of sentences and whether this was related to language skill.

Following our preregistered analysis plan, we examined activation during an auditory sentence processing task that included morphological errors of finiteness. The results of these preregistered analyses indicated that both phonological and semantic mechanisms were activated during processing of sentences with morphological errors. Phonological mechanisms, however, appeared especially involved as the results indicated activation within our phonological regions of interest in both the pSTG and dorsal IFG, compared to only activation in the ventral IFG, and not in the pMTG, in our semantic functional localizer. The cluster of activation in the dorsal IFG was especially large, spanning 242 voxels (see Table 5). One interpretation of these results is that morphological processing in sentences first engages the pSTG where phonological representations are stored before engaging both phonological and semantic mechanisms in the IFG to further access these representations. In other words, morphological processing may rely first on the dorsal stream for speech perception and only then also engage the ventral stream for further lexical processing. Given the large cluster in the dorsal IFG, phonological processing remains especially important, likely due to its role in segmentation of speech sounds. This is in line with the dual stream model of speech processing that assumes the STG stores phonological representations that can be mapped onto lexical representations in the temporal cortex or articulatory codes in the frontal cortex (Hickok and Poeppel 2007; Hickok 2022).

That phonological mechanisms seem to be particularly engaged for morphological processing is in accordance with theories of both general sentence processing and morphological processing. In theories of sentence processing, it is assumed that phonological processing occurs prior to semantic encoding (Friederici 2002; Friederici 2011); thus, to access the meaning of specific morphemes, these morphemes first need to be phonologically segmented. A similar argument is made in two main theories concerning morphological processing, the Distributed Morphology Framework and the Connectionist Morphology Framework, where word form, ie phonology, needs to be processed before semantic encoding of morphemes can take place. The current study design is not suited to provide direct evidence for one theory over the other as one main difference between these theories is whether morphemes at the word and sentence level are processed the same or differently, and our study only examined morphological processing in sentences.

Based on previous results (van der Kleij et al. 2019; Schwarz et al. 2023), we hypothesized that the extent to which phonological and semantic mechanisms were engaged during morphological processing might be related to one’s language abilities. To examine this, partial correlation analysis between activation during morphological processing of sentences and language abilities was conducted within our functional localizers. We expected that those with higher language abilities would rely on phonological mechanisms while those with lower language abilities would rely on semantic mechanisms. Our correlation analysis did not yield a significant relationship, indicating that there is no observable effect of language abilities on the neural basis of morphological processing within our functional localizers. As we only had limited variability in our language data (see Fig. 3), additional research in linguistically diverse samples is needed. It is also possible that there exist brain–behavior correlations outside of our regions of interest.

Exploratory whole-brain analysis examining the correlation between the neural basis of morphological processing and language abilities was conducted. The results showed a negative correlation in the cerebellum, indicating that children with lower language skills show higher activation in the cerebellum during morphological processing. Although the cerebellum has traditionally been related to motor planning and coordination (eg Stoodley and Schmahmann 2009; Rudolph et al. 2023), interest in the “linguistic cerebellum” has risen (for meta-analyses, see Vias and Dick 2017; Vlasova et al. 2023). Especially relevant here is the proposal that the cerebellum may play a role in phonological processing and in the amplification and refinement of these cortical representations (eg Booth et al. 2007). Indeed, verbal fluency studies have shown that cerebellar patients (either through degenerative disease or because of brain lesions) were impaired on phonological fluency tasks, but not on semantic fluency tasks (Leggio et al. 2000; Arasanz et al. 2012), indicating unique associations between the cerebellum and phonological processing. The engagement of the cerebellum by children with relatively lower language abilities may be compensatory in nature. It is possible that children with lower language skill have deficient phonological segmentation in the cortical language regions, and therefore engage the cerebellum to amplify and refine these representations to compensate. This could allow them to segment these morphemes in the service of sentence processing.

It is known that children with DLD experience challenges in the use of morphemes (eg Georgiou and Theodorou 2023). They especially experience difficulties with tense-related morphemes (eg Rice and Wexler 1996; Witherstone 2024), potentially because these morphemes have relatively low phonetic contrast and therefore require more processing effort (Montgomery and Leonard 2006). Previous language-focused fMRI examinations (eg during nonword repetition, passive listening, etc.) of children with DLD show mixed results, with some studies indicating reduced activation in the IFG and STG compared to neurotypical children and others not observing such differences (for a literature overview, see Abbott and Love 2023). Although a recent meta-analysis shows the only reliable difference in DLD is in the basal ganglia, there is an indication that there may be differences in the inferior frontal, superior temporal, and inferior parietal in this group (eg Lee et al. 2020; Bahar et al. 2024), areas that we show to be involved during morphological processing in this study. Notably, this meta-analysis shows little evidence for any alteration of the anterior cerebellum (Ullman et al. 2024). Future work should examine the neural pattern for morphological processing in children with DLD to determine whether their reduced ability to process morphology is due to alterations of phoneme segmentation in the superior temporal cortex that is compensated for by the engagement of the cerebellum.

Our results showing that phonological and semantic mechanisms in the frontal cortex are involved in morphological processing are in line with previous research suggesting that the frontal cortex shows specialization for phonological and semantic processing in children who are ≥7 years old while younger children show greater evidence of specialization in the temporal cortex (eg Weiss et al. 2018; Mathur et al. 2020; Wang et al. 2023). Therefore, it is possible that younger children engage different brain areas during morphological processing. We have preregistered an analysis plan to examine the same research question in 5-year-old children (https://osf.io/4wnr8). Based on developmental neurocognitive models of language processing that argue for a maturational gradient from the temporal to the frontal cortex and for earlier maturation of phonological compared to semantic mechanisms (Skeide and Friederici 2016), we might find evidence that higher language skill is related to reliance on phoneme segmentation in the superior temporal cortex during processing morphology in sentences.

## Conclusion

This project focused on the role of phonological and semantic mechanisms at the word level underlying morphological processing at the sentence level. The preregistered results of our fMRI study show that both phonological mechanisms in the pSTG and dorsal IFG and semantic mechanisms in ventral IFG, but not pMTG, are involved in morphological processing. The selective engagement of the superior temporal cortex and the large cluster engaged in the dorsal IFG suggests that phonological segmentation is particularly important for isolating morphemes in order to access their meaning. Although we did not find brain–behavior correlations with language skill in our planned comparisons in our region of interest, exploratory whole-brain analyses revealed that lower language abilities were associated with greater activation in the cerebellum during the morphological processing of sentences. It could be that cerebellar involvement is a compensatory mechanism for deficient phonological segmentation in the cortical structures.

## Data Availability

The data necessary to reproduce the analyses presented here are publicly accessible on OpenNeuro via https://openneuro.org/datasets/ds003604/versions/1.0.7. The analytic scripts necessary to reproduce the analyses presented in this paper can be found on GitHub (https://github.com/marjoleinmues/Phonological_Semantic_Mechanisms_During_Morphological_Processing_7yo). The analyses presented here were preregistered on Open Science Framework (https://osf.io/juzcm).
